# Yolk Sac Tumor in an Eight-Year-Old Girl: A Case Report and Literature Review

**DOI:** 10.3389/fped.2019.00169

**Published:** 2019-04-30

**Authors:** Li Hsun Chen, Kui-Chuen Yip, Hsing-Ju Wu, Su-Boon Yong

**Affiliations:** ^1^Asian Institute of Tele-surgery (IRCAD-Taiwan), Chang Bing Show Chwan Memorial Hospital, Lukang, Taiwan; ^2^Division of Family Medicine, Chang Bing Show Chwan Memorial Hospital, Lukang, Taiwan; ^3^Research Assistant Center, Show Chwan Memorial Hospital, Changhua, Taiwan; ^4^Department of Medical Research, Chang Bing Show Chwan Memorial Hospital, Lukang, Taiwan; ^5^Institute of Medicine, Chung Shan Medical University, Taichung, Taiwan; ^6^Division of Pediatric Allergy, Immunology and Rheumatology, Department of Pediatrics, Show Chwan Memorial Hospital, Changhua, Taiwan; ^7^Department of Nursing, Meiho University, Pingtung, Taiwan

**Keywords:** alpha-fetoprotein, chemotherapy, yolk sac tumor, pediatric, surgery

## Abstract

Yolk sac tumor (YST), which most frequently arises in the gonads as a type of germ cell tumor, is rare in children but is highly malignant. It has been suggested that alpha-fetoprotein (AFP) can be applied as a feasible tumor marker because its level was elevated in >90% of YST. The treatment generally involves debulking surgery of tumors followed by systemic chemotherapy. Metastasis process of YST in children is different from that in adults and thus the treatment option is required. In this study, we described a rare case of YST in terms of the clinical manifestation, imaging, and histopathology findings, diagnosis and treatment in an 8-year-old girl. Furthermore, it is important to investigate more thoroughly a patient with history of intermittent abdominal pain and fever with previously multiple accesses, because these might be the critical signs for YST that should be alarmed for early treatment. Although YST is rare in children, pediatric physicians should be aware of this and prompt treatment should be addressed.

## Introduction

Ovarian germ cell tumors (OGCTs) in which neoplasms form in the germ cells of the ovary, account for about 15~20% of all ovarian neoplasms (1). Only 1~2% of OGCTs are malignant called malignant ovarian germ cell tumors (MOGCTs) and constitute around 3–5% of all malignant ovarian neoplasms (2). Yolk sac tumor (YST) or endodermal primitive tumor accounting the second most common tumor in MOGCTs (3) is rare and typically occurs in gonads (4, 5). YST often presents in young women or adolescent girls with the ages between 18 and 30 years old; ~33% of YST patients are premenarchal (1, 6). In comparison with epithelial ovarian tumors, YST is highly malignant growing rapidly with a very brief duration of symptoms which metastasizes fast and intrudes all intra-abdominal structures and retroperitoneal lymph nodes (5, 7). YST was universally life-threatening before the development of combination chemotherapy. With the introduction of novel chemotherapeutic regimens in the end of 1970s, the 5-year survival rates of YST significantly improved from 14% to nearly 90% (8). Especially adding cisplatin to combination therapies, prognosis of the patients reached excellent values, even for patients with advanced stages (5). Therefore, YST is rare in children and malignant; however, it could be cured usually. In this study, we described a rare case of YST in an 8-year-old girl in terms of the clinical presentation, imaging findings, diagnosis, and treatment.

## Case Presentation

**Figure 1:**
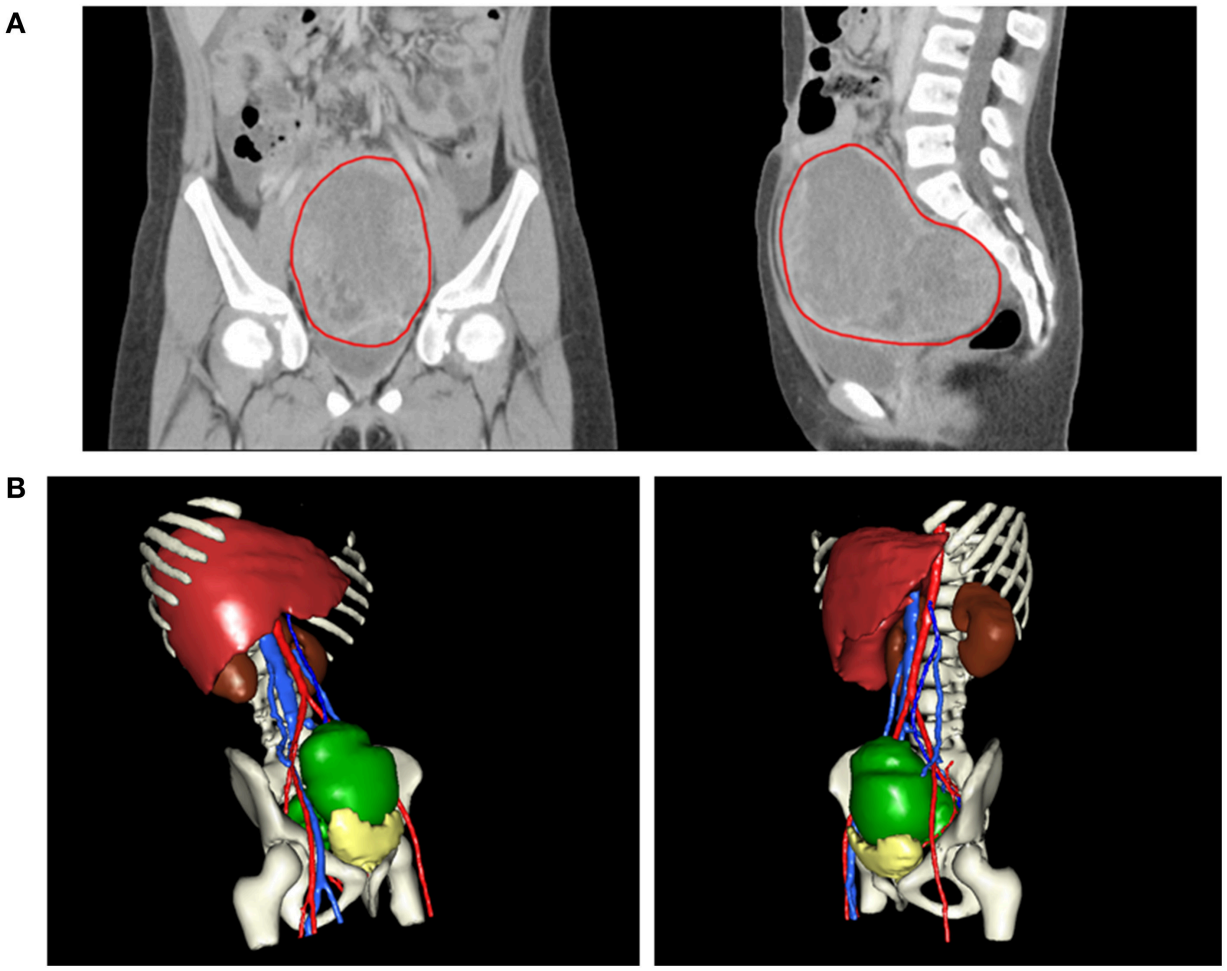
**(A)** CT revealing a large pelvic mass measuring 12 × 9.7 × 7.38 cm in size (circled by red line). **(B)** A three-dimensional virtual model created from 1.25-mm thin-slice CT images showing the tumor of 12 × 9.7 × 7.38 cm in size (green).

An 8-year-old girl suffered from intermittent abdominal pain and fever for 3 weeks. She had visited local clinics and regional hospital for several times. Oral medication was prescribed for pain and constipation, but her symptoms persisted. Then, she was brought to the Pediatrics outpatient department with nausea, vomiting and lower abdominal pain for 2 months. On physical examination, lower abdomen tenderness, and pale looking were noted. The ultrasonography (US) showed a huge pelvic tumor measuring 11.1 × 9.4 cm in size with cystic mass (3.6 × 2.8 cm) and bloody ascites over right subhepatic and lower abdominal area. There was no family history of cancer. The clinical diagnosis was pelvic mass, possibly neoplastic in nature. Computed tomography (CT) revealed a large pelvic mass, measuring 12 × 9.7 × 7.38 cm in size with internal heterogeneity and gynecological origin (ovary or urterus), enlarged paraaortic and mesenteric lymph nodes and fluid in Morrison pouch (Figure 1A). A three-dimensional (3D) virtual model was created from 1.25-mm thin-slice CT images and the green area illustrates the tumor (Figure 1B). The personal 3D model of patient's pelvic part may further improve the understanding of complex anatomy of the uterus, bladder and blood vessels. The values of tumor markers, alpha-fetoprotein (AFP), CA125 and beta human chorionic gonadotropin (hCG) were 13220.25 ng/ml, 536.7 U/ml, and <1.2 mIU/ml, respectively. The tumor was at the central pelvic cavity with direct invasion to the surrounding organs including the rectal and sigmoid colon walls, small bowel walls, the bladder wall and the cul-de-sac. In addition, it also involved the left para-adnexal tissues and the tip of appendix. Thus, the patient underwent debulking operation in which bilateral salpingo-oophorectomy, pelvic lymph node dissection, omentectomy, appendectomy, and radical excision of the implanted tumors were performed. Although the left ovary *per se* was grossly not involved by tumor, metastasis occurred as tumor invasion to the left periadnexal tissues and the left infundibulopelvic ligament were noted, thus left salpingo-oophorectomy was also done in order to prevent residual tumors and possible secondary surgery.

The patient was discharged 5 days after operation and recovered well. Gross examination of the specimen submitted for pathology showed a piece of necrotic tissue, measuring 3.5 × 2.0 × 0.3 cm in size. H&E stained sections of the specimen revealed tumor necrosis with small focus of atypical cells with hyperchromatic nuclei mainly in glandular or reticular pattern. Immunohistochemical staining revealed the residual cells were immunoreactive to AFP and glypican-3 (GPC-3). The findings were consistent with YST. The patient was then referred to the oncologist for further treatment and under stringent follow-up.

## Discussion

YST is a rare tumor of childhood, which account for ~3.5% of all childhood cancers (<15 years) and usually arises in gonads, testis or ovary, thus a type of germ cell tumor. The incidence between the ages 15 and 19 rises to 16% (9). Approximately one-third are extra-gonadal origins, such as vagina, mediastinum, pineal gland, cervix, endometrium, and sacrococcygeal area (9–12). It has also been reported that YST occurred in penile shaft, urachus, stomach, liver, lungs, heart, thyroid, nasal region, cranial base, vulva, retroperitoneum, prostate, pericardium, diaphragm, mesentery, mouth, ears, omentum, eyes, and subcutaneous region (13–30). Also, the recent YST case reports in children are summarized in Table 1. There are two types of MOGCTs, germinomatous and non-germinomatous (2, 34). YST are the commonest non-germinomatous MOGCTs (34). In children, the vast majority of YST (85%) presented as clinical stage I in comparison with 35% in adults (35).

**Table 1:** Summary of the reported cases for yolk sac tumor in children after 2010.

**No**.	**Age/gender**	**Initial symptoms**	**Diagnosis**	**Size and location of tumor**	**Alpha fetoprotein (AFP)**	**Treatment**	**Death**	**References**
1	12 months/female; 46 months/female	Vaginal bleeding; Vaginal bleeding	US, CT, MRI, exploratory laparotomy and histopathological analysis; Otoscope, US, MRI, vaginoscopy, cystoscopy and histopathological analysis	5 cm, 2 cm	34, 247 ng/ml; 4.4 ng/ml	Three cycles of PEB (cisplatin, etoposide, bleomycin) chemotherapy every 3 weeks. Thereafter, JEB (carboplatin, etoposide, and bleomycin) chemotherapy every 4 or 5 weeks. Surgery and four cycles of JEB chemotherapy every 3 or 4 weeks	No; No	(10)
2	9 months/female	Right-sided facial swelling	MRI, histopathological analysis, US, bone marrow examination, cerebrospinal fluid examination, and bone scan	3.2 × 3.0 × 5.6 cm, right masticator space	18,964 ng/ml	Surgery and four cycles of PEB chemotherapy	No	(25)
3	9 months/female	Intermittent fever, cough, and mild tachypnea	Chest X-ray, US, CT, laparotomy, and pathological analysis	10 × 10 × 7.4 cm, right upper abdomen, and right lower chest	10,055 ng/ml	Chemotherapy with eight cycles of carboplatin, etoposide, and bleomycin (the JEB regimen) every 3 weeks, surgery and 1 more cycle of chemotherapy with carboplatin, VP16, and bleomycin	No	(17)
4	5 years/male	A large distal penile shaft mass associated with multiple groin swellings	US and pathological analysis	Two masses of 10 × 8 × 6 cm and 6 × 6 × 4 cm, penile shaft	–	Surgery and three courses of PEB chemotherapy	Yes	(15)
5	10 years/female	An enlarging, non-painful right-sided neck mass	US, CT, histopathological analysis and flexible bronchoscopy	5.1 × 3.5 × 3.6 cm, right thyroid lobe	14,204.7 ng/ml	Right hemithyroidectomy and four 3-week cycles of PEB chemotherapy	No	(21)
6	14 months/female	A rapidly-growing mass in the floor of her mouth, dysphagia	MRI and histopathological analysis	3 × 4 cm, oral cavity above the geniohyoid muscles	–	Surgery	Yes	(23)
7	3 years/female	A swelling in the back	X-ray, US, histopathological analysis, CT, MRI, and bone scintigraphy	5 × 7 mm, left lumbar paravertebral subcutaneous region	278 IU/ml	Surgery and five cycles of PEB chemotherapy	No	(24)
8	2 years/male	Gradual progressive forward protrusion of the right eye	CT, MRI and histopathological analysis	superonasal quadrant of the right orbit	78.1 ng/ml	Surgery and four 3-week cycles of PEB chemotherapy	No	(26)
9	13 months/female	Hematoma on the upper lip	MRI, histopathological analysis, CT and US	4 × 5 cm, at the midline of the upper lip	>1,308 ng/ml	Two cycles of Adriamycin, vincristine, cyclophosphamide and cisplatin (AVCP) chemotherapy and 1 cycle of ifosfamide, etoposide, and vincristine (IEV) chemotherapy and surgery	No	(27)
10	18 months/male	Fever, cough, and breathlessness	CT and histopathological analysis	multiple hyperechoic nodules in both lung fields	9,322 ng/ml	Surgery and adjuvant chemotherapy	Yes	(28)
11	18 months/female	–	Histopathological analysis, MRI, and US	1.5 × 1.3 × 0.8 cm, right hypochondriac region of the abdominal wall connected to skin	58 ng/ml	Surgery	No	(31)
12	9 years/female	Abdominal distention with a dull aching pain	CT, exploratory laparotomy, and histopathological analysis	15 × 13 × 12 cm, left ovary	>1,000 ng/ml	Surgery (left salpingo-oophorectomy, omentectomy, appendicectomy with removal of the serosal deposits over the terminal ileum and sigmoid) and BEP chemotherapy	No	(32)
13	14 months/female	Intermittent blood in the diaper	US, cystoscopy, vaginoscopy, and MRI	3.6 × 2.3 × 2.5 cm, vagina	1,386 ng/ml	Four cycles of PEB chemotherapy	No	(12)
14	15 years/male	Swelling of his face and neck, dyspnea, cough with whitish sputum, dysphagia	Chest X-ray, CT, electrocardiogram (ECG), echocardiography, and histopathological analysis	15 × 12 cm, anterior mediastinum predominantly on the left hemithorax	> 8,000 ng/ml	Five fraction of radiotherapy (total of 1,500 cGy)	Yes	(29)
15	3 years/male	Melena, anemia, progressive abdominal distension and low-grade fever	Upper gastrointestinal endoscopy, CT, histopathological analysis, and laparotomy	7 × 8 cm, epigastrium	21,000 ng/ml	Three courses of 3 weekly PEB chemotherapy and sleeve gastrectomy with omentectomy	No	(30)
16	13 years/female	Deepening of the voice and amenorrhea	MRI and histopathological analysis	14.3 × 14.5 × 8.0 cm, displacing the front of the uterus	>1,210 ng/ml	Surgery: unilateral salpingo-oophorectomy and partial omentectomy and four courses of PEB chemotherapy	No	(33)
17	8 years/female	Intermittent abdominal pain and fever	US, CT, and histopathological analysis	12 × 9.7 × 7.38 cm, over right subhepatic and lower abdominal area	13,220.25 ng/ml	Debulking operation: bilateral salpingo-oophorectomy, pelvic lymph node dissection, omentectomy, appendectomy, and radical excision of the implanted tumors	No	Our case

There are a number of diagnostic tools applied for YST, such as US, CT, magnetic resonance imaging (MRI) and histopathological analysis (Table 1). US characterizes the adnexal mass and shows ascites or hepatic metastasis. CT scan detects carcinosis and adenopathy, MRI reveals the hyper-vascularized and hemorrhagic feature of the mass (36). Moreover, exploratory laparotomy is emerging as a tool to detect the details of the tumor and surrounding involvement and also for biopsy (10, 32). YST are heterogeneous with a number of different histolopathological subtypes. In newborns and younger children, YST are predominant variant, whereas there are a wide variety of subtypes in adolescent. The typical histopathological features of YST are solid, tubular and focal papillary patterns with Schiller-Duval bodies and sinusoidal structures with fibrovascular cores lining formed by tumor cells, frequent mitotic figures and are cytokeratinpositive (14, 31).

It has been suggested that AFP can be applied as a feasible tumor marker because its level was elevated in >90% of YST (37). In our case, the level of AFP was also increased (13220.25 ng/ml). Thus, the prognosis of patients can be monitored by the AFP level after operation. It has been reported that the lowering level of post-operative serum AFP could be an useful marker for determining if residual cancer cells still exists after surgery (38). The national comprehensive cancer network (2016) recommends that patients who completed clinical course are monitored by AFP every 2~4 months for 2 years after treatment (36). Also, response to chemotherapy could be assessed by the AFP level (34). In particular, a postoperative AFP level of >1,000 ng/ml could serve as a prognostic indicator for the ovarian YST patients (39). However, the studies suggest a slight increase in AFP should not be applied as the sole criterion for chemotherapy decision (40).

The exact pathogenesis of YST remains obscure. However, some studies propose that it occurs from malignant transformation of misplaced germ cells. During the 4–6th week of embryogenesis, germ cells migrate laterally between the embryonic ectoderm and endoderm; germ cell tumor can arise anywhere along the migration from the mesoderm to the future cranial area (4). Although the pathogenesis of extragonadal germ cell tumors is unclear, two possible explanations were proposed: (1) misintegration of germ cells during development of embryo and (2) distribution of germs cells to other organs (41). Therefore, more research is required for investigating the mechanism of pathogenesis in order to develop for effective treatment for YST.

The general treatment for YST is surgery for eliminating the primary tumor without severe morbidity (34) (Table 1). In patients with stage I YST, previous studies proved that treatment of adnexectomy showed equivalent results to extensive surgery (8, 42). The treatment of OGCTs in the advanced stage generally involves debulking surgery of tumors followed by adjuvant chemotherapy (7) (Table 1). Several studies support the regimen of BEP (bleomycin, etoposide, and cisplatin) for primary treatment of the OGCT patients (36). They demonstrated a significantly high 5-year survival rate of 94%, even for the patients with residual cancers (42–44). The national comprehensive cancer network recommends 3–4 BEP cycles after surgical resection (36). Furthermore, it was reported that platinum-based chemotherapy should be used for the more malignant tumors such as endodermal sinus tumor and mixed germ cell tumor (45, 46). Neoadjuvant chemotherapy could be considered for the patients having extensive intra-abdominal disorders when initial surgical debulking is not preferred (47). Chemotherapy is suggested for treating recurrence (37). BEP chemotherapy is considered as a gold-standard first-line treatment for germ cell tumors at all stages (48). An important issue to consider for treating young patients is to reserve fertility by using fertility sparing strategy. Fertility-sparing surgery is possible to achieve because most tumors are unilateral (34). Furthermore, minimally invasive surgery has been proved to have better prognosis (34). Rudaitis et al. (47) applied neoadjuvant chemotherapy of four BEP cycles to decrease the tumor size in order to minimize the extent of surgery and thus the impact on the patient's fertility. In spite of the risks of damaging the reproductive function of female patients involved in chemotherapy, previous investigations have revealed that most of the women could recover their normal menstrual and reproductive functions post treatment (49, 50). After treatment, follow-ups are required such as abdominal and pelvic examination, CT, chest X-ray and AFP levels (51). Furthermore, YST metastasizes through the hematogenous route in >50% of the pediatric patients in comparison with only 4–6% of the adult patients (52). This fact modifies the therapeutic strategy. Thus, Retroperitoneal Lymph Node Dissection (RPLND) would not be the appropriate treatment for pediatric patients and complications such as wound infection, atelectasis, pulmonary insufficiency secondary to bleomycin-induced interstitial fibrosis, chylous ascities, small bowel obstruction, and subsequently ejaculatory dysfunction could occur (37). Herein, we also provided our experience in managing YST in children.

## Conclusion

Our case highlights the importance of YST in children, and we provided our valuable experiences in the approaches of diagnosis and treatment for YST in children. Furthermore, it is important to investigate more thoroughly a patient with history of intermittent abdominal pain and fever with previously multiple accesses, because these might be the critical signs for YST that should not be neglected in order to treat the patient earlier. Although YST is rare in children, pediatric physicians should be still aware of this as it can be fatal and prompt treatment should be addressed.

## Ethics Statement

We have obtained verbal and written informed consent from the patient's guardian for the publication of this case report.

## Author Contributions

LC, K-CY, and S-BY wrote the manuscript. H-JW reviewed and revised the manuscript. All authors approved the final version of this manuscript.

### Conflict of Interest Statement

The authors declare that the research was conducted in the absence of any commercial or financial relationships that could be construed as a potential conflict of interest.
